# Role of Physical Activity on Mental Health and Well-Being: A Review

**DOI:** 10.7759/cureus.33475

**Published:** 2023-01-07

**Authors:** Aditya Mahindru, Pradeep Patil, Varun Agrawal

**Affiliations:** 1 Department of Psychiatry, Jawaharlal Nehru Medical College, Datta Meghe Institute of Medical Sciences, Wardha, IND

**Keywords:** anxiety, depression, morbidity, mental health, physical activity

## Abstract

In addition to the apparent physical health benefits, physical activity also affects mental health positively. Physically inactive individuals have been reported to have higher rates of morbidity and healthcare expenditures. Commonly, exercise therapy is recommended to combat these challenges and preserve mental wellness. According to empirical investigations, physical activity is positively associated with certain mental health traits. In nonclinical investigations, the most significant effects of physical exercise have been on self-concept and body image. An attempt to review the current understanding of the physiological and psychological mechanisms by which exercise improves mental health is presented in this review article. Regular physical activity improves the functioning of the hypothalamus-pituitary-adrenal axis. Depression and anxiety appear to be influenced by physical exercise, but to a smaller extent in the population than in clinical patients. Numerous hypotheses attempt to explain the connection between physical fitness and mental wellness. Physical activity was shown to help with sleep and improve various psychiatric disorders. Exercise in general is associated with a better mood and improved quality of life. Physical exercise and yoga may help in the management of cravings for substances, especially in people who may not have access to other forms of therapy. Evidence suggests that increased physical activity can help attenuate some psychotic symptoms and treat medical comorbidities that accompany psychotic disorders. The dearth of literature in the Indian context also indicated that more research was needed to evaluate and implement interventions for physical activity tailored to the Indian context.

## Introduction and background

Physical activity has its origins in ancient history. It is thought that the Indus Valley civilization created the foundation of modern yoga in approximately 3000 B.C. during the early Bronze Age [1]. The beneficial role of physical activity in healthy living and preventing and managing health disorders is well documented in the literature. Physical activity provides various significant health benefits. Mechanical stress and repeated exposure to gravitational forces created by frequent physical exercise increase a variety of characteristics, including physical strength, endurance, bone mineral density, and neuromusculoskeletal fitness, all of which contribute to a functional and independent existence. Exercise, defined as planned, systematic, and repetitive physical activity, enhances athletic performance by improving body composition, fitness, and motor abilities [2]. The function of physical activity in preventing a wide range of chronic illnesses and premature mortality has been extensively examined and studied. Adequate evidence links medical conditions such as cardiovascular disease and individual lifestyle behaviours, particularly exercise [3]. Regular exercise lowered the incidence of cardiometabolic illness, breast and colon cancer, and osteoporosis [4]. In addition to improving the quality of life for those with nonpsychiatric diseases such as peripheral artery occlusive disease and fibromyalgia, regular physical activity may help alleviate the discomforts of these particular diseases [5]. Exercise also helps with various substance use disorders, such as reducing or quitting smoking. As physical exercise strongly impacts health, worldwide standards prescribe a weekly allowance of "150 minutes" of modest to vigorous physical exercise in clinical and non-clinical populations [6]. When these recommendations are followed, many chronic diseases can be reduced by 20%-30%. Furthermore, thorough evaluations of global studies have discovered that a small amount of physical exercise is sufficient to provide health benefits [7].

## Review

Methodology

In this review article, a current understanding of the underlying physiological and psychological processes during exercise or physical activity that are implicated in improving mental health is presented. Search terms like "exercise" or "physical activity" and "mental health", "exercise" or "physical activity" and "depression", "exercise" or "physical activity" and "stress", "exercise" or "physical activity" and "anxiety", "exercise" or "physical activity" and "psychosis," "exercise" or "physical activity" and "addiction" were used as search terms in PubMed, Google Scholar, and Medline. An overwhelming majority of references come from works published within the past decade.

The impact of physical health on mental health

There is an increasing amount of evidence documenting the beneficial impacts of physical activity on mental health, with studies examining the effects of both brief bouts of exercise and more extended periods of activity. Systematic evaluations have indicated better outcomes for mental diseases with physical activity. Numerous psychological effects, such as self-esteem, cognitive function, mood, depression, and quality of life, have been studied [8]. According to general results, exercise enhances mood and self-esteem while decreasing stress tendencies, a factor known to aggravate mental and physical diseases [9]. Studies show that people who exercise regularly have a better frame of mind. However, it should be highlighted that a consistent link between mood enhancement and exercise in healthy individuals has not been established.

**Figure 1:**
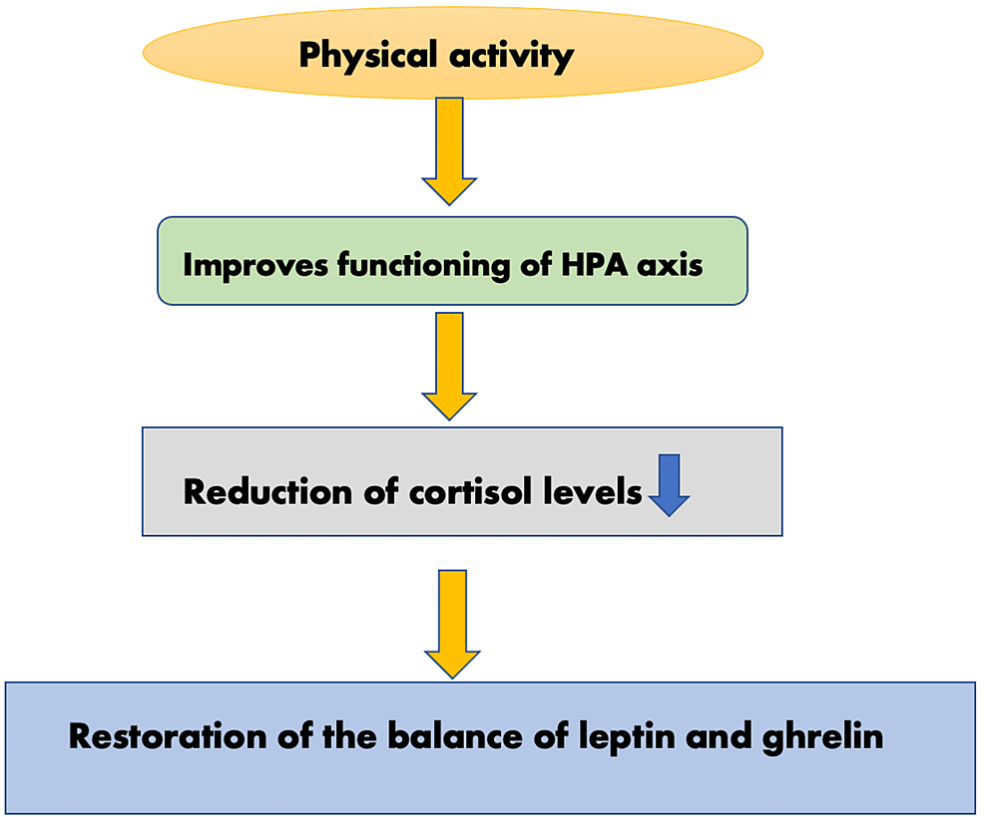
The effects of physical activity on the HPA axis HPA: hypothalamus-pituitary-adrenal This image has been created by the authors.

Additionally, human beings produce more of these two neurochemicals when they engage in physical activity. Human bodies manufacture opioids and endocannabinoids that are linked to pleasure, anxiolytic effects, sleepiness, and reduced pain sensitivity [10]. It has been shown that exercise can improve attention, focus, memory, cognition, language fluency, and decision-making for up to two hours [11]. Researchers state that regular physical activity improves the functioning of the hypothalamus-pituitary-adrenal (HPA) axis, lowering cortisol secretion and restoring the balance of leptin and ghrelin (Figure 1) [12].

Regular exercise has immunomodulatory effects such as optimising catecholamine, lowering cortisol levels, and lowering systemic inflammation. Physical activity has been shown to increase plasma brain-derived neurotrophic factor (BDNF), which is thought to reduce amyloid-beta toxicity linked to Alzheimer's disease progression [13].

Although no causal correlations have been proven, methodologically sound research has discovered a related improvement in mentally and physically ill populations. These findings are based on research and studies conducted all across the globe, particularly in the Western Hemisphere. In order to address a widespread health problem in India, it is useful to do a literature review that draws on research conducted in a variety of settings. In addition, the prevalence of these mental illnesses and the benefits of exercise as a complementary therapy might be made clear by a meta-analysis of research undertaken in India [14].

This review also analysed published literature from India to understand the effects of exercise on mental health and the implications for disease management and treatment in the Indian context. Results from Indian studies were consistent with those found in global meta-analyses. The Indian government has made public data on interventions, such as the effects of different amounts of physical exercise. Exercising and yoga have been shown to be effective adjunct therapies for a variety of mental health conditions [12]. Though yoga may not require a lot of effort to perform, other aspects of the program, such as breathing or relaxation exercises, may have an impact on a practitioner's mental health at the same time. Due to its cultural significance as a common physical practice among Indians and its low to moderate activity level, yoga would be an appropriate activity for this assessment [15].

Yoga as an adjunctive treatment 

Although yoga is a centuries-old Hindu practice, its possible therapeutic effects have recently been studied in the West. Mind-body approaches have been the subject of a lot of studies, and some of the findings suggest they may aid with mental health issues on the neurosis spectrum. As defined by the National Center for Complementary and Alternative Medicine, "mind-body interventions" aim to increase the mind's potential to alter bodily functions [16]. Due to its beneficial effects on the mind-body connection, yoga is used as a treatment for a wide range of conditions. Possible therapeutic benefits of yoga include the activation of antagonistic neuromuscular systems, stimulation of the limbic system, and a reduction in sympathetic tone.

Anxiety and depression sufferers might benefit from practising yoga. Yoga is generally safe for most people and seldom causes unintended negative consequences. Adding yoga to traditional treatment for mental health issues may be beneficial. Many of the studies on yoga included meditation as an integral part of their methodology. Meditation and other forms of focused mental practice may set off a physiological reaction known as the relaxation response. Functional imaging has been used to implicate certain regions of the brain that show activity during meditation. According to a wealth of anatomical and neurochemical evidence, meditation has been shown to have far-reaching physiological effects, including changes in attention and autonomic nervous system modulation [17]. Left anterior brain activity, which is associated with happiness, was shown to rise considerably during meditation. There's also some evidence that meditation might worsen psychosis by elevating dopamine levels [18-20]. We do not yet know enough about the possible downsides of meditation for patients with mental illness, since this research lacks randomised controlled trials.

Physical activity and schizophrenia

Schizophrenia is a debilitating mental disorder that often manifests in one's early years of productive life (late second decade). Remission of this disorder occurs in just a small fraction of cases. More than 60% will have relapses, and they might occur with or without noticeable deficits. Apart from delusions, hallucinations, and formal thought disorders, many patients exhibit cognitive deficits that emerge in the early stages of the disease and do not respond adequately to therapy [21].

Treatment for schizophrenia is challenging to master. Extrapyramidal side effects are a problem with first-generation antipsychotic drugs. Obesity and dyslipidemia have been related to second-generation drugs, which may cause or exacerbate these conditions. The majority of patients do not achieve complete remission, and many do not even experience satisfactory symptom relief. Even though certain antipsychotic medications may alleviate or even exacerbate negative and cognitive symptoms, these responses are far less common. This means that patients may benefit from cognitive rehabilitation. Because of their illness or a negative reaction to their medicine, they may also have depressive symptoms. This would make their condition even more disabling. Many patients also deal with clinical and emotional complications. Tardive extrapyramidal illnesses, metabolic syndromes, defect states, and attempted suicide are all in this category. Patient compliance with treatment plans is often poor. The caregivers take on a lot of stress and often get exhausted as a result.

Evidence suggests that increased physical activity can aid in attenuating some psychotic symptoms and treating medical comorbidities that accompany psychotic disorders, particularly those subject to the metabolic adverse effects of antipsychotics. Physically inactive people with mental disorders have increased morbidity and healthcare costs. Exercise solutions are commonly recommended to counteract these difficulties and maintain mental and physical wellness [22].

The failure of current medications to effectively treat schizophrenia and the lack of improvement in cognitive or negative symptoms with just medication is an argument in favour of utilising yoga as a complementary therapy for schizophrenia. Even without concomitant medication therapy, co-occurring psychosis and obesity, or metabolic syndrome, are possible. The endocrine and reproductive systems of drug abusers undergo subtle alterations. Numerous studies have shown that yoga may improve endocrine function, leading to improvements in weight management, cognitive performance, and menstrual regularity, among other benefits. In this context, the role of yoga in the treatment of schizophrenia has been conceptualized. However, yoga has only been studied for its potential efficacy as a therapy in a tiny number of studies. There might be several reasons for this. To begin with, many yoga academies frown against the practice being adapted into a medical modality. The second misconception is that people with schizophrenia cannot benefit from the mental and physical aspects of yoga practised in the ways that are recommended. Third, scientists may be hesitant to recommend yoga to these patients because of their lack of knowledge and treatment compliance.

In a randomised controlled experiment with a yoga group (n = 21) and an exercise group (n = 20), the yoga group exhibited a statistically significant reduction in negative symptoms [2]. In accordance with the most recent recommendations of the National Institute for Health and Care Excellence (NICE), the above research provides substantial evidence for the use of yoga in the treatment of schizophrenia. According to a meta-analysis of 17 distinct studies [23] on the subject, frequent physical activity reduces the negative symptoms associated with schizophrenia considerably.

Physical activity and alcohol dependence syndrome

Substance abuse, namely alcohol abuse, may have devastating effects on a person's mental and physical health. Tolerance and an inability to control drinking are some hallmarks of alcoholism. Research shows that physical activity is an effective supplement in the fight against alcohol use disorder. In addition to perhaps acting centrally on the neurotransmitter systems, physical exercise may mitigate the deleterious health consequences of drinking. Evidence suggests that persons with alcohol use disorder are not physically active and have low cardiorespiratory fitness. A wide number of medical comorbidities, like diabetes mellitus, hypertension, and other cardiovascular illnesses, occur with alcohol use disorders. Physical exercise may be highly useful in aiding the management of these comorbidities [24].

Physical exercise and yoga may help in the management of cravings for substances when other forms of therapy, such as counselling or medication for craving management are not feasible or acceptable. Physical exercise has been shown to have beneficial effects on mental health, relieve stress, and provide an enjoyable replacement for the substance. However, the patient must take an active role in physical activity-based therapies rather than passively accept the process as it is, which is in stark contrast to the approach used by conventional medicine. Since most substance use patients lack motivation and commitment to change, it is recommended that physical activity-based therapies be supplemented with therapies focusing on motivation to change to maximise therapeutic outcomes.

One hundred seventeen persons with alcohol use disorder participated in a single-arm, exploratory trial that involved a 12-minute fitness test using a cycle ergometer as an intervention. Statistically, significantly fewer cravings were experienced by 40% [24]. Exercise programmes were found to significantly reduce alcohol intake and binge drinking in people with alcohol use disorder in a meta-analysis and comprehensive review of the effects of such therapies [25].

Physical activity and sleep

Despite widespread agreement that they should prioritise their health by making time for exercise and sufficient sleep, many individuals fail to do so. Sleep deprivation has negative impacts on immune system function, mood, glucose metabolism, and cognitive ability. Slumber is a glycogenetic process that replenishes glucose storage in neurons, in contrast to the waking state, which is organised for the recurrent breakdown of glycogen. Considering these findings, it seems that sleep has endocrine effects on the brain that are unrelated to the hormonal control of metabolism and waste clearance at the cellular level. Several factors have been proposed as potential triggers for this chain reaction: changes in core body temperature, cytokine concentrations, energy expenditure and metabolic rate, central nervous system fatigue, mood, and anxiety symptoms, heart rate and heart rate variability, growth hormone and brain-derived neurotrophic factor secretion, fitness level, and body composition [26].

After 12 weeks of fitness training, one study indicated that both the quantity and quality of sleep in adolescents improved. Studies using polysomnography indicated that regular exercise lowered NREM stage N1 (very light sleep) and raised REM sleep (and REM sleep continuity and performance) [22]. As people age, both short- and long-term activities have increasingly deleterious effects on sleep. In general, both short- and long-term exercise were found to have a favourable effect on sleep quality; however, the degree of this benefit varied substantially among different sleep components. On measures of sleep quality, including total sleep time, slow-wave sleep, sleep onset latency, and REM sleep reduction, acute exercise had no effect. But both moderate and strenuous exercise has been shown to increase sleep quality [27]. According to a meta-analysis of randomised controlled trials, exercise has shown a statistically significant effect on sleep quality in adults with mental illness [28]. These findings emphasise the importance that exercise plays in improving outcomes for people suffering from mental illnesses.

Physical activity in depressive and anxiety disorders

**Figure 2:**
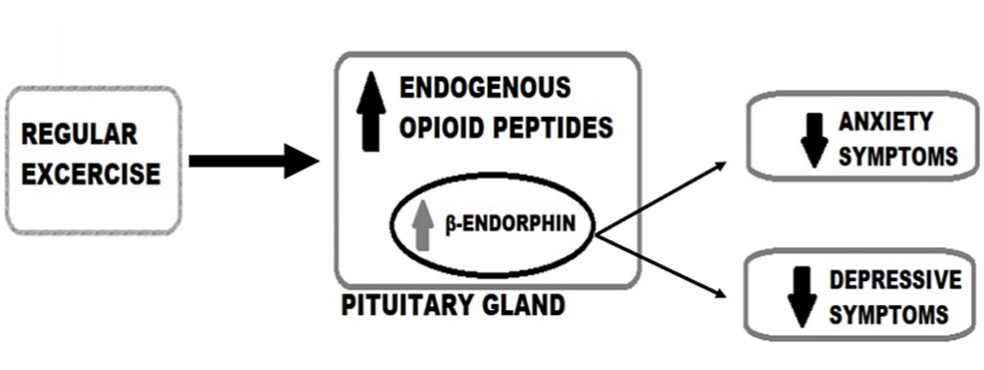
Flow chart demonstrating the link between beta-endorphins and depressive and anxiety symptoms This image has been created by the authors.

Depression is the leading cause of disability worldwide and is a major contributor to the global burden of disease, as per the World Health Organization. However, only 10%-25% of depressed people actually seek therapy, maybe due to a lack of money, a lack of trained doctors, or the stigma associated with depression [29]. For those with less severe forms of mental illness, such as depression and anxiety, regular physical exercise may be a crucial part of their treatment and management. Exercise and physical activity might improve depressive symptoms in a way that is comparable to, if not more effective than, traditional antidepressants. However, research connecting exercise to a decreased risk of depression has not been analysed in depth [30]. Endorphins, like opiates, are opioid polypeptide compounds produced by the hypothalamus-pituitary system in vertebrates in response to extreme physical exertion, emotional arousal, or physical pain. The opioid system may mediate analgesia, social bonding, and depression due to the link between b-endorphins and depressive symptoms (Figure 2).

The "endorphin hypothesis" states that physical activity causes the brain to produce more endogenous opioid peptides, which reduce pain and boost mood. The latter reduces feelings of worry and hopelessness. A recent study that demonstrated endorphins favourably improved mood during exercise, and provided support for these theories suggested that further research into the endorphin theory is required [31].

Physical activity and exercise have been shown to improve depressive symptoms and overall mood in people of all ages. Exercise has been implicated in lowering depressive and anxious symptoms in children and adolescents as well [32]. Pooled research worldwide has revealed that physical exercise is more effective than a control group and is a viable remedy for depression [33]. Most forms of yoga that start with a focus on breathing exercises, self-awareness, and relaxation techniques have a positive effect on depression and well-being [34]. Despite claims that exercise boosts mood, the optimal kind or amount of exercise required to have this effect remains unclear and seems to depend on a number of factors [35].

Exercise as a therapy for unipolar depression was studied in a meta-analysis of 23 randomised controlled trials involving 977 subjects. The effect of exercise on depression was small and not statistically significant at follow-up, although it was moderate in the initial setting. When compared to no intervention, the effect size of exercise was large and significant, and when compared to normal care, it was moderate but still noteworthy [36]. A systematic evaluation of randomised controlled trials evaluating exercise therapies for anxiety disorders indicated that exercise appeared useful as an adjuvant treatment for anxiety disorders but was less effective than antidepressant treatment [37].

## Conclusions

The effects of exercise on mental health have been shown to be beneficial. Among persons with schizophrenia, yoga was shown to have more positive effects with exercise when compared with no intervention. Consistent physical activity may also improve sleep quality significantly. Patients with alcohol dependence syndrome benefit from a combination of medical therapy and regular exercise since it motivates them to battle addiction by decreasing the craving. There is also adequate evidence to suggest that physical exercise improves depressive and anxiety symptoms. Translating the evidence of the benefits of physical exercise on mental health into clinical practice is of paramount importance. Future implications of this include developing a structured exercise therapy and training professionals to deliver it. The dearth of literature in the Indian context also indicates that more research is required to evaluate and implement interventions involving physical activity that is tailored to the Indian context.
